# Early surgery to prevent embolic events in patients with infective endocarditis: a comprehensive review

**DOI:** 10.1186/s13019-024-02946-x

**Published:** 2024-07-22

**Authors:** Sikander Tajik Nielsen, Katra Hadji-Turdeghal, Peter Laursen Graversen, Lauge Østergaard, Morten Holdgaard Smerup, Lars Køber, Emil Loldrup Fosbøl

**Affiliations:** 1 Department of Cardiology, Copenhagen University Hospital – Rigshospitalet, Blegdamsvej 9, Copenhagen, 2100 Denmark; 2 Department of Clinical Medicine, University of Copenhagen, Blegdamsvej 3B, Copenhagen, 2200 Denmark; 3 Department of Cardiothoracic Surgery, Copenhagen University Hospital – Rigshospitalet, Blegdamsvej 9, Copenhagen, 2100 Denmark

**Keywords:** Endocarditis, Surgery, Embolism, Valve, Heart

## Abstract

**Background:**

Infective endocarditis (IE) is a dangerous and lethal illness with high mortality rates. One of the main indications for surgery according to the guidelines is prevention of embolic events. However, uncertainty remains concerning the timing of surgery and the effect of early surgery in combination with antibiotic therapy versus antibiotic therapy alone in IE patients with a vegetation size > 10 mm.

**Methods:**

We conducted a comprehensive review by searching the PubMed, MEDLINE, and EMbase databases. Titles and abstracts were screened, and studies of interest were selected for full-text assessment. Studies were selected for review if they met the criteria of comparing surgical treatment + antibiotic therapy to antibiotic therapy alone in patients with vegetations > 10 mm.

**Results:**

We found 1,503 studies through our database search; nine of these were eligible for review, with a total number of 3,565 patients. Median age was 66 years (range: 17–80) and the median percentage of male patients was 65.6% (range: 61.8 − 71.4%). There was one randomised controlled trial, one prospective study, and seven retrospective studies. Seven studies found surgery + antibiotic therapy to be associated with better outcomes in patients with IE and vegetations > 10 mm, one of them being the randomised trial [hazard ratio = 0.10; 95% confidence interval 0.01–0.82]. Two studies found surgery + antibiotic therapy was associated with poorer outcomes compared with antibiotic therapy alone.

**Conclusion:**

Overall, data vary in quality due to low numbers and selection bias. Evidence is conflicting, yet suggest that surgery + antibiotic therapy is associated with better outcomes in patients with IE and vegetations > 10 mm for prevention of emboli. Properly powered randomised trials are warranted.

## Background

Infective endocarditis (IE) is a significant lethal illness with high mortality rates despite advances in both medical and surgical treatments [1–3]. In-hospital mortality is 15–25% [4] and 1-year mortality rate is about 30% [5, 6]. The standard treatment includes long-term antibiotic therapy or a combination of surgery and antibiotic therapy in some cases. Surgery + antibiotic therapy is chosen in 25–50% of cases [7, 8]. One of the three main indications for IE surgery is prevention of embolic events [9, 10], which is a feared and serious complication in patients with IE. The incidence of systemic embolization in patients with IE ranges from 10 to 50% [10–13], and especially cerebral embolization is associated with high mortality rates [10, 13].

Both the European Society of Cardiology (ESC) and American College of Cardiology/American Heart Association (ACC/AHA) have developed guidelines providing recommendations for the proper timing of surgery in patients at risk of developing emboli [9, 10, 14]. However, the level of evidence (LOE) is low. The previous ESC IE guidelines from 2015 recommended surgical treatment within days in case of vegetation exceeding 15 mm and no prior embolic events with a class IIB recommendation and LOE C [9]. Similarly, the ACC and AHA recommend surgery during initial hospitalization and before completion of full antibiotic treatment with a class IIb, LOE B. In contrast, the ESC 2023 IE guidelines recommendations have lowered the vegetation size to 10 mm or above with no embolic events and low surgical risk with a class IIb, LOE B. The new guidelines define early surgical treatment as: “within 3–5 days”, primarily based on a study conducted in 2012 [2].

The aim of this comprehensive review is to elucidate the evidence on early surgical treatment in combination with antibiotic therapy for preventing embolic events in IE patients with vegetations > 10 mm, compared to antibiotic therapy alone.

## Methods

We conducted a comprehensive review by searching the PubMed, MEDLINE, and EMbase databases. For the PubMed database, we used the following search strings: (“Endocarditis/surgery“[Mesh] OR “Endocarditis/therapy“[Mesh]) and vegetation. For the MEDLINE and EMbase databases the following search strings: ((“endocarditis” and “surgery”) OR (“endocarditis” and “therapy”) AND vegetation NOT case report) were used. Additionally, relevant studies were identified by examining the reference sections of studies deemed eligible for review. The final search was done on the 24th of October 2023.

### Inclusion and exclusion criteria

Studies had to be either randomised controlled trials (RCT), or retrospective or prospective observational studies. Further, the studies had to compare surgical + antibiotic treatment to antibiotic therapy alone in the context of embolic risk reduction. Studies that compared early surgery + antibiotic therapy to antibiotic therapy alone in the context of other indications for surgery were therefore not included. Similarly, studies that analysed prevention of emboli in IE patients, but did not include any comparison of surgical treatment were also excluded. Due to differences in treatment strategies, studies including right-sided IE were excluded. Only original articles written in English were included. Lastly, because of the change in population from young patients with rheumatic fever towards an older population with multiple comorbidities, articles had to be published after January 1st, 2000.

### Study selection

**Fig. 1:**
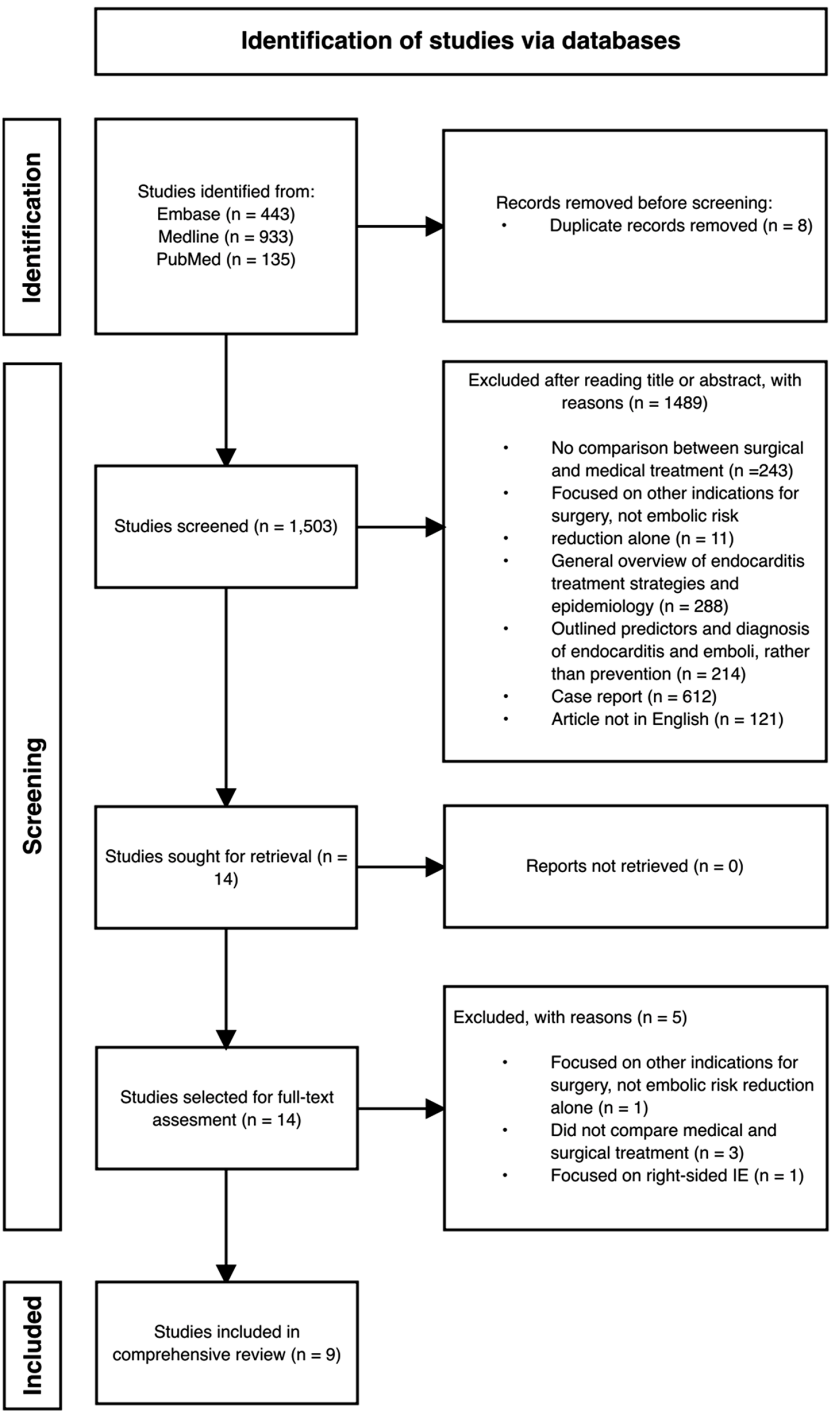
Flowchart illustrating the selection process. IE = Infective endocarditis

A single reviewer conducted a title and abstract screening, followed by a full-text screen for articles with relevance. Discrepancies were discussed and resolved with three other reviewers. Figure 1 illustrates the selection of included studies.

## Results

Our search strings identified a total of 1,503 citations. Of these, nine studies were included. Among the selected studies were seven retrospective studies, one prospective study, and one RCT. Table 1 summarises the 2015 and 2023 ESC guidelines’ recommendations for surgery to prevent emboli.

**Table 1:** Comparison between the 2015 and 2023 ESC guidelines’ recommendations for surgery to prevent emboli [9, 14]

ESC guideline	Surgical indication	Class of recommendation	Level of evidence
2015^a^			
	“Aortic or mitral NVE or PVE with persistent vegetations > 10 mm after one or more embolic episode despite appropriate antibiotic therapy”	I	B
	“Aortic of mitral NVE or PVE with isolated large vegetations (> 15 mm) and no other indication for surgery”	IIb	C
	“Aortic or mitral NVE with vegetations > 10 mm, associated with severe valve stenosis or regurgitation, and low operative risk”	IIa	B
	“Aortic or mitral NVE or PVE with isolated very large vegetations (> 30 mm)”	IIa	B
2023			
	“Urgent surgery^b^ is recommended in aortic or mitral NVE or PVE with persistent vegetations ≥ 10 mm after one or more embolic episode despite appropriate antibiotic therapy”	I	B
	“Urgent surgery is recommended in IE with vegetation ≥ 10 mm and other indications for surgery”	I	C
	“Urgent surgery may be considered in aortic or mitral IE with vegetation ≥ 10 mm and without severe valve dysfunction or without clinical evidence of embolism and low surgical risk”	IIb	B

ESC = European Society of Cardiology; NVE = Native valve endocarditis; PVE = Prosthetic valve endocarditis; IE = Infective endocarditis

^a^All 3 indications are recommended performed “within a few days” in the 2015 ESC guideline

^b^Within 3–5 days

### Study characteristics

The total number of patients from the nine studies was 3,565 and the median number of patients was 355 (range: 71–1,006). The median age was 66 years (range: 17–80) and the median percentage of male patients was 65.6% (range: 61.8 − 71.4%). The causative microorganisms were identified in 2,416 patients (67.8%); the most frequently found were: Viridans group streptococci (33.2%), *Staphylococcus aureus* (28.5%), Enterococci (16.8%).

Three studies were multinational, three were national ones collecting data from multiple tertiary centres, and the remaining three were single centre studies. All nine studies focused on left-sided IE only. A single study examined IE in patients with prosthetic valves, the remaining eight analysed patients with native valve IE.

All nine studies divided their populations into patients treated with surgery + antibiotic therapy and patients managed with antibiotic therapy alone. The eight cohort studies retrieved patient data from either international databases, or from electronic medical records. To even out differences in baseline characteristics and compare clinical outcomes, propensity score matching was performed in five studies [15–19]. Five studies subdivided patients based on vegetation size (> 10 mm vs. <10 mm) [15, 16, 18, 20, 21]. Three studies identified patients who underwent surgery with embolic risk reduction as the only surgical indication [19, 22]. The RCT randomised patients with vegetations > 10 mm to surgery + antibiotic therapy or antibiotic therapy alone. The one study investigating prosthetic valve IE [17] compared surgery + antibiotic therapy to antibiotic therapy alone, regardless of vegetation size.

### Outcomes

Five studies [16, 18, 21–23] evaluated long-term (+ 5 years) survival outcomes between the surgery + antibiotic therapy groups and the antibiotic therapy alone groups, four also included the occurrence of embolism after being admitted to a tertiary centre as well as recurrence of IE. Two studies [15, 20] analysed 6-month mortality, one study reported 90-day mortality [19], and one on in-hospital mortality [15]. Table 2 summarises the results of the included articles.

**Table 2:** Summary of articles reviewed

Author	Location	Population size	Study design	Percentage operated on	Intervention/ exposure	Endpoint(s)	Results	Main limitations
Kang et al. [2]	Seoul, South Korea	76	RCT	48.7%	Early surgery vs. conventional therapy to prevent emboli in IE patients with vegetations > 10 mm.	Death, embolic event, or recurrence of IE within 6 weeks and 7 years.	Primary endpoint reached in 3% vs. 23%, respectively. 7-year follow-up revealed event-free survival of 87% vs. 59%, respectively.	Patients of lower risk and mean age (47y) much lower than overall IE-patient population. Small population size.
Fosbøl et al. [19]	Multinational	1,006	Retrospective register study using ICE-Plus database, propensity matching used.	53.4%	Vegetation size and surgery’s effect on 6-month mortality.	In-hospital or 6-month mortality.	Larger vegetation size carries higher mortality. Mortality after surgery is not affected by vegetation size.	Lack of data regarding cause of death in cohort. All ICE centres are tertiary centres, possibility of selection and referral bias.
Kim et al. [20]	Seoul, South Korea	132	Prospective enrolment from 1998–2006, with 44 pairs propensity matched.	48.5%	Effect of early surgery in prevention of emboli, compared to antibiotic therapy alone in patients with IE.	Embolism, cardiovascular mortality, recurrence of IE.	Higher 5-year survival rate, and lower percentage reaching endpoint in early surgery group [HR 0.14 (95% CI 0.03–0.64)].	Early surgery patients tended to have larger vegetations. Propensity matching not sufficient to completely avoid confounding.
Wang et al. [17]	Multinational	148	Retrospective register study using ICE-MD database, propensity matching used.	42.0%	Mortality in patients with PVIE who underwent surgery, compared to similar patients who did not undergo surgery.	In-hospital mortality.	Lower in-hospital mortality and increased survival in surgically treated group [OR 0.56 (95% CI 0.23–1.36)].	Certain variables were incomplete. Propensity matching not sufficient to completely avoid confounding.
Young et al. [20]	London, United Kingdom	142	Retrospective study using electronic patient records. Propensity matching was not used.	69.0%	Association between vegetation size on valve destruction, embolism and mortality.	6-month mortality.	Vegetation area was associated with mortality in antibiotic therapy alone group (HR 1.01 [95% CI 1.00–1.02, p-value < 0.01], but not in surgery + antibiotic therapy group.	Selection and referral bias, due to retrospective design at tertiary centre.
Song et al. [21]	Seoul, South Korea	419	Retrospective study using electronic patient records. Propensity matching was not used.	65.2%	Association between vegetation size and survival and cerebral emboli.	Long-term mortality.	Vegetation size was only associated with mortality in patients treated with antibiotic therapy alone. Surgical treatment was strongly associated with better 1-, 3-, and 5- year survival (97.4% vs. 89.7% and 96.3% vs. 85.6% and 95.4% vs. 83.3%) and lower risk of embolism (OR 0.78 (0.5–1.14)).	Selection bias. Unknown if patients had already suffered cerebral embolism before echo.
Scheggi et al. [18]	Multinational	638	Retrospective study using electronic patient records. 49 surgical patients propensity matched with 98 patients treated with antibiotic therapy alone.	33.3%	All-cause mortality in patients with prevention of embolism as only surgical indication.	5-year survival.	Surgically managed patients have lower mortality rates, compared to patients treated with antibiotic therapy alone [HR 0.223 (95% CI 0.079–0.656)]. 5-year survival rate higher in surgical patients (82.4% vs. 66.5%)	Changes in management of IE during long study period. Tertiary centre with lower surgical mortality than smaller centres.
Desch et al. [22]	Luebeck, Germany	71	Observational study. All patients diagnosed with IE at single centre between 2000 and 2012 included. Propensity matching was not used.	83.1%	All-cause mortality in patients with prevention of embolism as only surgical indication.	Long-term mortality.	Surgery was independently associated with mortality, compared to antibiotic therapy alone [HR 3.9 (95% CI 0.9–16 − 6)]	Selection and scientific bias. Physicians possibly tended to operate on higher-risk patients. Small sample size.
Cabezón et al. [19]	Madrid, Spain	726	Retrospective study using electronic patient records. 70 surgically treated patients matched with 69 patients treated with antibiotic therapy alone.	50.4%	All-cause mortality in patients with prevention of embolism as only surgical indication.	90-day mortality.	Surgically treated with no other indications for surgery had lower survival probability than patients treated with antibiotic therapy alone (81.4% vs. 88.4%).	Selection and referral bias, due to retrospective design at tertiary centre.

RCT = Randomized controlled trial; IE = Infective endocarditis; ICE = International Collaboration on Endocarditis; HR = Hazard ratio; CI = Confidence interval

### Comparison of embolic rates

Three studies [2, 16, 21] compared the rate of embolism after admission between the surgery + antibiotic therapy groups and the antibiotic therapy alone groups. In the Kang et al. study, eight patients (21%) in the antibiotic therapy alone group suffered systemic emboli within six weeks compared to zero in the surgery + antibiotic therapy group. Further, eight patients (21%) form the antibiotic therapy alone group suffered a systemic embolism within six months. Again, no patients from the surgery + antibiotic therapy group suffered a systemic embolism within six months. Kim et al. conducted a similar evaluation where 14 patients in the antibiotic therapy alone group suffered a systemic embolism during initial hospitalization, compared to zero in the surgery + antibiotic therapy group. During follow-up, two patients from each group had suffered a systemic embolism. Song et al. evaluated the rate of systemic embolism after initial hospitalization. 49 (33.6%) patients from the antibiotic therapy alone group suffered a systemic embolism, compared to 77 (28.2%) in the surgery + antibiotic therapy group. None of the studies statistically tested if surgery was associated with a significant risk reduction of systemic emboli.

### Other complications

Two studies [2, 16] evaluated the recurrence of IE. In both studies, one patient from the antibiotic therapy alone group was readmitted with IE, and none from the surgery + antibiotic therapy group.

### Mortality

Of the nine studies included in this review, seven indicate that surgery is beneficial to short- and long-term mortality. This includes the RCT, where in-hospital mortality or embolism was significantly lower in the surgery + antibiotic therapy group, compared to the antibiotic therapy alone group [hazard ratio (HR) = 0.10; 95% confidence interval (CI) 0.01–0.82.]. Further, the long-term survival rates at 7-year follow-up were also better in the surgery + antibiotic therapy group (87% vs. 83%) as was the event-free survival rate (87% vs. 59%) [23]. The largest study in this review [15] (1,006 patients) found larger vegetation sizes to be associated with higher mortality rates, both in-hospital [Odds ratio (OR) 1.45 (95% CI 1.04–2.03)] and 6-months after admission [OR 1.37 (CI 95% 1.01–1.87)]. However, in the surgery + antibiotic therapy group, vegetation size was not associated with a higher mortality rate at either endpoint [OR 1.01 (95% CI 0.69–1.49)]. This study also propensity matched patients using Cox regression to eliminate confounders. When comparing patients with vegetations > 10 mm, the surgically treated patients had significantly higher survival probability compared to the patients treated with antibiotic therapy alone. Another study [20] examining vegetation sizes provided similar results. Vegetation area > 50 mm^2^ was only associated with mortality in the antibiotic therapy alone group. A fourth study [18] propensity matched 147 patients (49 receiving surgery + antibiotic therapy vs. 98 receiving antibiotic therapy alone) who had prevention of embolism as the only surgical indication. The surgery + antibiotic therapy group had lower mortality rates [HR 0.22 (95% CI 0.08–0.66)] and better estimated 5-year Kaplan-Meier survival rates (95% CI): 82.4% (71.9 − 94.4%) for surgically treated patients vs. 66.5% (57–77.6%) in patients treated with antibiotic therapy alone. Three retrospective database studies [16, 17, 21] also found that surgery + antibiotic therapy, in patients with vegetations > 10 mm, improved either long-term survival rates, in-hospital mortality rates, or both.

The remaining two studies found that surgery + antibiotic therapy, in patients with embolic risk reduction as the only surgical indication, is not beneficial when compared to antibiotic therapy alone (210 patients). The single-centre study identified 71 patients with no surgical indications other than prevention of embolism where 59 patients underwent surgery. Using Cox regression, the study found that surgery was independently associated with increased mortality rates [HR 3.9 (95% CI 0.9–16.6)]. The second study was able to identify 70 patients who underwent surgery with no other indication, and 69 who only received antibiotic therapy alone. The surgery + antibiotic therapy group had a 90-day survival probability of 81.4% vs. 88.4% in the antibiotic therapy alone group.

## Discussion

This study aimed to examine the current evidence on the effectiveness of early surgical therapy to prevent embolic events in patients with IE, when compared to antibiotic treatment alone. A total of nine studies were included: one RCT, one prospective study, and seven retrospective studies. Seven studies indicate that surgery + antibiotic therapy, as opposed to antibiotic therapy alone, reduces mortality in IE patients that are at risk of developing emboli. A total of three studies showed lower rates of emboli in surgically treated patients. The remaining two studies found evidence of the contrary; surgical treatment was associated with either higher mortality rates or lower probability of survival.

Kang et al.’s [2] RCT demonstrated the benefit of surgery within 48 h of admission in patients with vegetations > 10 mm, as fewer patients suffered death or emboli in the early surgery group. However, the population size was small, and despite it being an RCT, it is difficult to extrapolate data analysis from only 76 patients to the whole IE population. Furthermore, the study had many exclusion criteria resulting in the scope of the study being somewhat narrow. Patients included had a lower operative risk than usual, meaning that these results cannot be transferred to all patients with IE. Further, 77% of patients who were allocated in the conservative care group required surgical therapy during initial admission or during follow-up.

Both Fosbøl et al. [15] and Young et al. [20] indicate that surgery + antibiotic therapy during initial hospitalization was associated with better survival rates in patients with large vegetations and at risk of suffering embolic events. High mortality rates and complications were shown for patients with vegetations > 10 mm who received antibiotic therapy alone. However, Young et al. showed that for patients who underwent surgery, vegetation size and area was not associated with increased mortality. Further, Fosbøl et al. found patients with large vegetations, who did undergo surgery had the same 6-month mortality rates as patients with smaller vegetations. With emboli being one of the main complications in patients with large vegetations, results from this study indicate that surgery + antibiotic therapy has a positive effect on the prevention of this complication. Despite Fosbøl et al.’s study not being an RCT, propensity analysis was used to analyse surgery and vegetations size’s effect on mortality, and to minimize selection and survival bias. However, with the ICE-Plus registry being a collaboration of tertiary centres only, Fosbøl et al.’s study is still subject to selection and referral bias. This results in patients differing from the general population, and patients may be referred due to better chances of surviving a surgery.

Four other non-RCT studies also used propensity analysis. Three of these studies showed similar results in their propensity matched groups compared to the other studies analysed. Both Scheggi et al. [18] and Cabezón et al. [19] were able to identify patients, within their respective cohorts, undergoing surgery with prevention of embolism as sole indication for surgery. The results of the two studies were, however, significantly different. While Scheggi et al. identified surgically managed patients to have lower mortality rates and higher long-term survival rates, results from Cabezón et al.’s study indicated the opposite. Patients in this study who underwent surgery had lower survival probability than the patients treated with antibiotic therapy alone. The only other study with similar results as Cabezón et al. was the observational study by Desch et al. [22].

These contradicting results further add to the uncertainty of how this complicated disease is best managed. More RCTs are required in the future to help resolve this uncertainty [3]. Reasons for the lack of RCTs have been mainly ethical and logistical [2], but one could argue that we are at a point where the need for high quality data outweighs these concerns. That in fact, the actual ethical problem is that we are making clinical decisions based on low quality evidence, due to the lack of RCT data. Prevention of embolism remains the least frequent cause for operating patients with IE because of the lack of quality evidence [24]. It is important to note, that early surgery alone is not sufficient to prevent embolic events, as appropriate antibiotic therapy within the first week also reduces the risk of stroke [9, 13, 20]. The correlation between large vegetations and death has been established for a long time, but emboli as the cause of death in these situations remains unclear. The majority of evidence analysed in this study suggests that early surgery minimizes the risk of both emboli and death in patients with large vegetations, but there are still two studies contradicting these findings. While this evidence should not be ignored, the change toward a more aggressive surgical approach in the revised ESC guideline is interesting due to the fact of rather few studies on this subject. Therefore, the change in recommendations towards a more surgically aggressive approach can be interpreted somewhat controversial considering the levels of evidence remain at B and C. A recent POET sub-study [25] found the outcome of patients with IE and vegetations > 10 mm to be of no difference when compared to patients with smaller vegetations. However, this study only included patients who were in a stable condition. Further, the number of patients with strokes before randomization was higher in the group with larger vegetations. These patients were also more likely to have undergone cardiac surgery before randomization [OR: 7.86 (95% CI 4.57–13.49)].

Certain causative microorganisms can increase risk of embolic events and influence the decision to choose surgery over antibiotic therapy alone. However, the influence of specific microorganisms on both embolic events and mortality has not been statistically examined by the studies in our review.

### Study limitations

This review was conducted through a thorough search of large number of studies using three major databases. Almost all included studies were found in all three database searches. However, there are some limitations to this study. First, this review only includes nine studies, while this is a severe limitation, it also highlights the need for more studies within this field. With the majority of the included studies being retrospective studies from tertiary centres both selection and referral bias of some degree is inevitable. These biases might result in mortality rates being better in the surgical groups, as the patients with the most severe cases of IE are sometimes too sick to be offered surgical treatment. Further, although many studies used propensity matching as an effective way to reduce certain biases, it cannot eliminate the effect of confounding. Most of the studies included patients with IE according to the modified Duke criteria, however Kim et al. [16] and Wang et al. [17] included according to the Duke criteria.

This review only focused on studies that analysed vegetation size, but we believe multiple factors, such as mobility, contribute to poor patient outcomes. Future studies can potentially provide significant information on other vegetation parameters.

Finally, our article search was carried out by a single reviewer. While this increases the risk of relevant articles being missed, we attempted to reduce this risk for this by also screening references in all articles that were selected.

## Conclusion

In conclusion, the results from this review indicate surgical intervention in patients with IE, and vegetations > 10 mm, to be associated with a reduced risk of emboli, and short- and long-term risk of mortality. However, the underlying evidence is conflicting. The quality of the studies is limited due to size, case selection, and generalizability. A definitive conclusion on who and when to operate cannot be drawn upon this level of evidence. RCTs are therefore needed and are essential to provide high quality evidence.

### Perspectives

There are currently two ongoing RCTs comparing early surgery to conventional treatment. The Danish ASTERIx trial, and the French CHIRURGENDO trial. ASTERIx aims to enrol 496 patients and perform surgery within 48 h of randomization compared with antibiotic therapy, whereas CHIRURGENDO aims to enrol 208 patients with half of them undergoing surgery within 72 h of randomization and the other group receiving conventional therapy according to guidelines. Hopefully with the results from these large RCT’s soon, we will be able assess the effectiveness of early surgical therapy to prevent embolic events in patients with IE with intermediate-length vegetations (10–15 mm).

## Data Availability

No datasets were generated or analysed during the current study.

## Abbreviations

IE: Infective endocarditis
ESC: European Society of Cardiology
ACC/AHA: American College of Cardiology/American Heart Association
LOE: Level of evidence
RCT: Randomized controlled trials
HR: Hazard ratio
CI: Confidence interval
OR: Odds ratio
